# Deep cascaded registration and weakly-supervised segmentation of fetal brain MRI

**DOI:** 10.1016/j.heliyon.2024.e40148

**Published:** 2024-11-19

**Authors:** Valentin Comte, Mireia Alenya, Andrea Urru, Judith Recober, Ayako Nakaki, Francesca Crovetto, Oscar Camara, Eduard Gratacós, Elisenda Eixarch, Fatima Crispi, Gemma Piella, Mario Ceresa, Miguel A. González Ballester

**Affiliations:** a BCN MedTech, Department of Information and Communication Technologies, Universitat Pompeu Fabra, Barcelona, Spain; b BCNatal, Fetal Medicine Research Center (Hospital Clínic and Hospital Sant Joan de Déu), University of Barcelona, Barcelona, Spain; c Institut d’Investigacions Biomédiques August Pi i Sunyer (IDIBAPS), Barcelona, Spain; d Centre for Biomedical Research on Rare Diseases (CIBERER), Barcelona, Spain; e ICREA, Barcelona, Spain; f European Commission, Joint Research Centre (JRC), Ispra, Italy; g European Commission, Joint Research Centre (JRC), Geel, Belgium

**Keywords:** Registration, Segmentation, Cascade, Deep learning, Fetal brain

## Abstract

Deformable image registration is a cornerstone of many medical image analysis applications, particularly in the context of fetal brain magnetic resonance imaging (MRI), where precise registration is essential for studying the rapidly evolving fetal brain during pregnancy and potentially identifying neurodevelopmental abnormalities. While deep learning has become the leading approach for medical image registration, traditional convolutional neural networks (CNNs) often fall short in capturing fine image details due to their bias toward low spatial frequencies. To address this challenge, we introduce a deep learning registration framework comprising multiple cascaded convolutional networks. These networks predict a series of incremental deformation fields that transform the moving image at various spatial frequency levels, ensuring accurate alignment with the fixed image. This multi-resolution approach allows for a more accurate and detailed registration process, capturing both coarse and fine image structures. Our method outperforms existing state-of-the-art techniques, including other multi-resolution strategies, by a substantial margin. Furthermore, we integrate our registration method into a multi-atlas segmentation pipeline and showcase its competitive performance compared to nnU-Net, achieved using only a small subset of annotated images as atlases. This approach is particularly valuable in the context of fetal brain MRI, where annotated datasets are limited. Our pipeline for registration and multi-atlas segmentation is publicly available at https://github.com/ValBcn/CasReg.

## 1 Introduction

Fetal brain Magnetic Resonance Imaging (MRI) is an important asset in the early diagnosis of neurodevelopment abnormalities, such as those derived from Intrauterine Growth Restriction, Corpus Callosum Agenesis, or Ventriculomegaly [[1], [2], [3], [4], [5]]. In comparison with the adult brain, acquiring fetal brain MRI is a challenging procedure both at a clinical and technical level. A major difficulty is the appearance of motion artifacts caused by the fetal movements and the mother's breathing. To tackle this issue, snapshot imaging techniques, such as T2-weighted Single Shot Fast Spin Echo, are preferred to 3D volumetric imaging. These techniques produce low-resolution images with minimized motion artifacts that can be acquired along all planes. 3D Super-Resolution Reconstruction (SRR) algorithms, such as proposed by Ebner et al. [6] or Kuklisova-Murgasova et al. [7], are then applied tocombine the low resolution images and form high resolution 3D volumes. Once they are reconstructed, the analysis of fetal brain MRI involves the application of advanced techniques, including image registration and multi-class tissue segmentation. Image registration, which consists of aligning a pair of images, serves as a critical tool for gaining insights into the evolution of brain structures throughout the course of pregnancy. It can be used to model the fetal brain development and help clinicians to identify potential neurodevelopmental abnormalities. Similarly, the accurate annotation of fetal brain tissues using multi-class segmentation is critical to characterize and quantify the rapidly evolving morphological features of the fetal brain.

## 2 Related works

Classical registration methods use intensity-based similarity metrics, such as cross-correlation (CC), mutual information, or sum of square distance, to measure the degree of alignment between the fixed image and the warped moving image, and maximize the similarity metric by iterative optimization methods. These methods are effective, but can be computationally intensive and time-consuming. In recent years, deep learning (DL)-based registration methods have been proposed as a faster alternative to classical methods, achieving similar results in a shorter amount of time. Most of these DL-based approaches update their parameters using a loss function based on the aforementioned intensity-based similarity metrics, and an additional regularization term to promote smooth and reversible transformation. DL has also revolutionized segmentation methods, excelling in both segmentation accuracy and computational efficiency. Numerous DL-based techniques for medical image segmentation have been proposed, often leveraging convolutional neural networks (CNNs) with an encoder-decoder structure, such as U-Net [8]. These approaches take advantage of the deconvolution concept [9] and incorporate skip connections between layers of the same dimension on either side of the network in order to preserve fine details in the feature maps. Many of the DL-based registration methods proposed in the recent years employ spatial transformer networks [10], introducing differentiable spatial transformation layers to generate dense deformation fields (DFs). For example, Li and Fan [11] presented a self-supervised method for registering pairs of 3D brain MRI scans, differing from traditional methods by eliminating network training. It relies on a Fully Convolutional Network optimized using Normalized Cross-Correlation (NCC) and a total variation term for DF regularization. Balakrishnan et al. [12] presented VoxelMorph, a CNN autoencoder with a UNet-like structure, featuring ReLU activation and skip connection between the feature maps. They showed the efficiency of their methods on 3D brain MRI scans, yielding superior results compared to traditional methods [13] in a much shorter amount of time. In the same trend, Chen et al. [14] introduced TransMorph, a hybrid CNN-Transformer model, replacing traditional convolutional blocks with Swin Transformer blocks. An original approach was proposed by Krebs et al. [15] to bypass traditional regularization techniques. They utilized a conditional variational autoencoder to learn low-dimensional probabilistic deformations from the latent space representations of images, and converted them into DFs using an exponentiation layer. Within this landscape, the Deep Learning Image Registration (DLIR) framework, authored by [16], emerged as an innovative unsupervised approach for training CNNs in medical image registration tasks. They performed B-Spline registration with transposed convolutions, reducing the memory cost and speeding up the registration process, and used a bending energy penalty to regularize the DF. Another key innovation of their methods is the fact that they introduced a multi-stage CNNs for registration. Their model starts with a first network for affine registration followed by several CNNs for deformable registration, with different grid dimension of the B-Spline in order to perform coarse-to-fine registration. With the same inspiration, Zhao et al. [17] proposed recursive cascaded networks. Their model consists of several cascaded networks, trained simultaneously, that warp progressively the moving image into the fixed image. They further enforced a synergy between the cascaded networks by computing the loss function only on the final output of the model.

Similarly, Wang et al. [18] proposed an unsupervised end-to-end recursive cascaded parallel network for image registration. Their method includes a combination unit that merges the warped image with the moving image at each iteration. However, these cascaded approaches do not incorporate a multi-resolution strategy, which would enable the model to process images at varying spatial resolutions for more accurate alignment. Such an approach was proposed by Zhu et al. [19] introduced an identity-mapping cascaded network for fMRI registration. In their model, multi-resolution processing is enforced by employing parallel branches of progressively lower resolution as the cascaded networks proceed. An overview of DL-based cascaded registration approaches is given in Table 1.

**Table 1:** Overview of state-of-the-art DL cascaded registration methods for medical imaging, including image modality, region of interest, use of a multi-resolution approach, and code availability.

Reference	Image modality	Region of interest	Multi-resolution	Code available
De Vos et al. (2019)	MRI, CT	Heart, Lungs	Yes	Yes
Zhao et al. [17]	MRI, CT	Brain, Liver	No	Yes
Lara et al.[39]	CT	Cardiac	No	No
Zhu et al. [19]	fMRI	Brain	No	No
Hu et al. [40]	MRI	Brain	Yes	No
Wang et al. [18]	MRI	Brain	Yes	No
Cai et al. [41]	CT	Liver	Yes	No

Atlas-based image segmentation, supported by registration techniques, was regarded as the most accurate and reliable method for medical image segmentation before the rise of DL-based segmentation. Early atlas-based image segmentation techniques involved aligning a single atlas with the target image through the use of registration. This was primarily due to the limited availability of manually annotated images and to the high computational cost of registration at the time. Once the atlas was registered with the target image, the resulting transformation was used to propagate the atlas segmentation labels onto the target image [[20], [21], [22]]. However, a single registration may not encompass all the anatomical variation from one brain to another. In this context, MAS gained popularity thanks to the contributions of researchers such as Rohlfing et al. [23], Klein et al. [24], and Heckemann et al. [25]. MAS employs multiple atlases, or reference images, which are registered to the target image to guide the segmentation process. Manually annotated labels from the atlases are then transferred onto the fixed image and combined using label fusion techniques to obtain a refined segmentation. Crucially, the registration process in MAS is regularized, which produces smoother and more invertible transformation, addressing potential topological inconsistencies that might arise with some automatic segmentation methods. Rohlfing et al. [23] were the first to apply multiple registrations followed by label fusion techniques for the segmentation of three-dimensional microscopy images of bee's brains. Their work demonstrated that MAS was far superior to single atlas-based segmentation. Building on this research, Klein et al. [24] showed the effectiveness of MAS for segmenting human brain MRI scans. Their results indicated that using a larger number of atlases in MAS can help capture a wider range of anatomical variability and improve segmentation accuracy. Typically, intensity-based registration tools such as Avants et al. [13], Klein et al. [26], or Rueckert et al. [27] are used to compute one independent registration between each atlas and the target. In the context of perinatal brain segmentation, Urru et al. [28] exploited the idea first proposed by Wang et al. [29] of pre-computed registration: rather than registering the image to segment with all the atlases, their pipeline solely registers the image with a common template that has been computational time. Despite its accuracy, MAS has some notable drawbacks, including extensive computational time, as multiple registrations are time-consuming using classical methods. However, the potential of applying DL-based registration techniques within the framework of MAS remains relatively unexplored, despite their capacity to speed up the registration process, significantly improve the segmentation performances, and enable unsupervised or weakly supervised segmentation, thanks to the use of annotated atlases. This presents a distinct advantage over DL-segmentation methods [8,30], which are typically supervised and rely heavily on large amounts of annotated data. Our proposed method improves upon existing cascaded registration techniques by utilizing a model that reduces information loss through the accumulation of the DFs produced by the cascades, rather than relying on a composition of successive transformations. The model processes the image at various resolutions, going from low spatial frequencies for the first cascade, towards higher spatial frequencies moving forward through the cascades. This multi-resolution is further enforced by a multi-scale image similarity loss that simultaneously computes Normalized Cross Correlation (NCC) for various patches sizes, allowing the model to focus both coarse and fine image details at different scales. Moreover, we go beyond existing DL registration works by implementing a derived MAS approach and demonstrate its competitive performances compared to nnU-Net [30]. The proposed MAS method, being inherently a weakly-supervised segmentation methodology, by its capability to segment images using only a small subset of annotated images as atlases, offers a considerable advantage over supervised DL segmentation techniques, which usually require large annotated training sets.

## 3 Materials and methods

### 3.1 Cascaded registration

In mathematical terms, the process of registering two 3D images can be expressed as follows: let $X_{mv}$ and $X_{fx}$ be the moving and fixed image, of size H × W × L, defined over a 3-dimensional domain $\Omega \subset R^{3}$. The goal of registration is to align the moving image with the fixed image using a spatial transformation $\varphi :R^{3}\to R^{3}$. The warped moving image $X_{wp}$ that is generated by this transformation should be as similar as possible to the fixed image $X_{fx}$:

(1) $$X_{wp}=X_{mv}\circ \varphi \approx X_{fx}$$

**Fig. 1:**
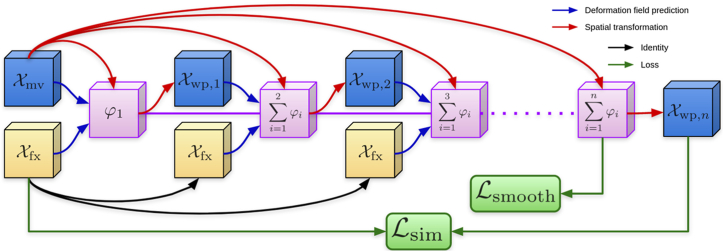

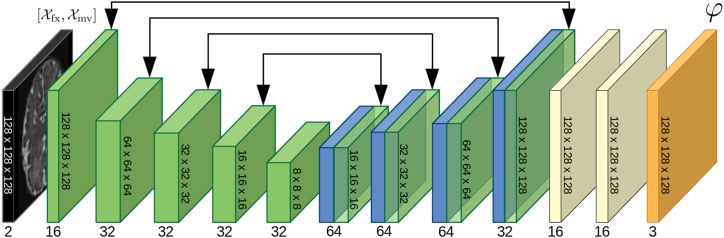

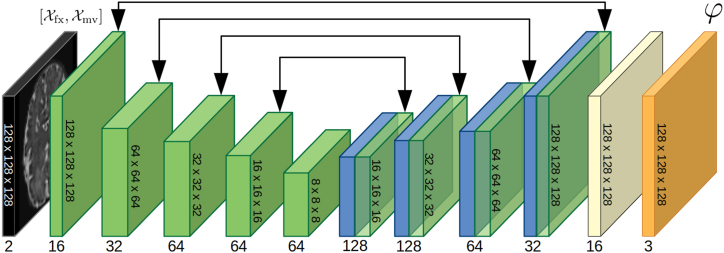
**(a)** The first partial DF φ1 is predicted using the fixed and moving images (blue arrows), and applied to the moving image in order to form the first warped image Xwp,1 (red arrows). Subsequently, the warped and fixed images are used to predict the second partial DF φ2 (blue arrows), which is summed with φ1 and applied to the moving image to form the second warped image (red arrows). This recursive pattern continues throughout the rest of the model **(b)** VoxelMorph architecture. (b) The proposed contracted architecture.

**Fig. 2:**
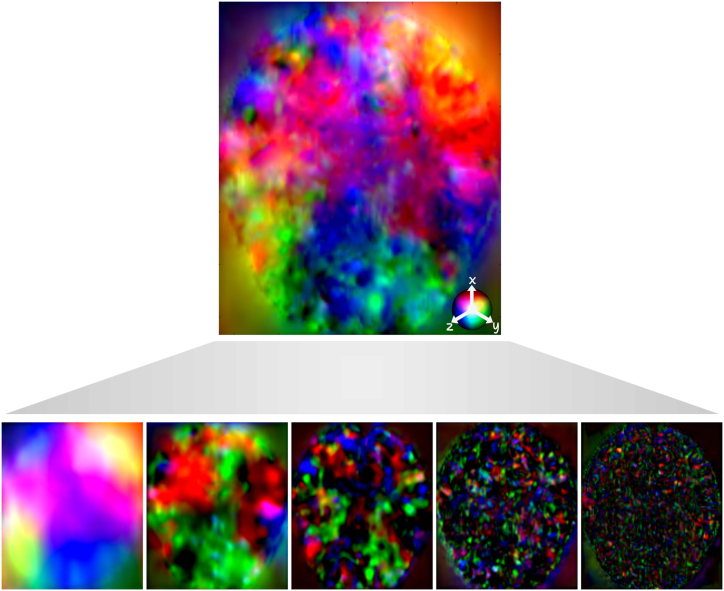
Illustration of the successive partial DFs produced by the cascaded networks (bottom) and the final DF (top). The color mapping is done by translating the three components of the deformation into RGB colors. We observe the increasing spatial frequency of the DFs, processing different levels of details.

Executing this procedure in a sequential manner to optimize the registration process aligns with the traditional paradigm of image registration, which often comprises multiple stages. These stages typically start with affine registration and then progress through a series of refinement steps in deformable image registration, following a coarse-to-fine strategy. This hierarchical multi-stage approach has been empirically shown to improve conventional iterative image registration [31]. In the domain of DL-based registration, a similar approach has been put into practice, as demonstrated by [16]. They proposed a multi-stage registration training utilizing stacked CNNs with varying grid sizes for B-Spline registration, enabling progressive and refined registration. In contrast [17], addresses this limitation by stacking multiple CNNs and training them concurrently. They compute the image similarity previously registered with all the atlases. This approach requires only one registration, which significantly reduces loss only on the final output, encouraging a collaborative synergy among the cascaded networks. In our present work, we build on this idea of collaborating cascaded networks. However, instead of progressively warping the moving image, our model accumulates the successive DFs generated by the cascades. This approach minimizes information loss caused by repeated interpolations. Our cascaded registration model decomposes the DF into smaller and simpler transformations, enabling image processing at various spatial frequencies. Fig. 1a illustrates the functioning of our cascaded model. The first network takes the moving image $X_{mv}$ and fixed image $X_{fx}$ as inputs and produces a dense DF, $\varphi_{1}$, which partially aligns the moving image with the fixed image. Subsequently, this first warped image, ${X_{wp}}_{0}$, is fed to the second network, together with $X_{fx}$, to generate $\varphi_{2}$, which is then summed with $\varphi_{1}$ towarp $X_{mv}$ into ${X_{wp}}_{1}$. This process is repeated with successive networks in a recursive manner, resulting in the final DF that fully aligns the moving image with the fixed image (Fig. 2):

(2) $${X_{\text{wp}}}_{n}=X_{\text{mv}}\;◦\sum_{i=1}^{n}\varphi_{i}.$$

For this work, we employ a loss function derived from Balakrishnan et al. [12], which is comprised of two main components: a similarity loss and a regularization loss. The similarity loss quantifies the degree of alignment between the warped image Xwp and the fixed image Xfx. This component is critical, as it drives the registration process to ensure that the two images are closely matched. We utilize negative local cross-correlation (NCC) as the measure for similarity, formulated as:

(3) $$L_{sim}=-CC\left(X_{fx},X_{wp}\right)=-\sum_{p\in \Omega }\;\frac{{\left(\sum_{p_{i}}\left(X_{fx}\left(p_{i}\right)-{\bar{X}}_{fx}\left(p\right)\right)\left(X_{wp}\left(p_{i}\right)-{\bar{X}}_{wp}\left(p\right)\right)\right)}^{2}}{\sum_{p_{i}}{\left(X_{fx}\left(p_{i}\right)-{\bar{X}}_{fx}\left(p\right)\right)}^{2}\cdot \;\sum_{p_{i}}{\left(X_{wp}\left(p_{i}\right)-{\bar{X}}_{wp}\left(p\right)\right)}^{2}\;}\text{,}$$

where $p_{i}$ iterates over the volume $d^{3}$ around the voxel p, ${\bar{X}}_{fx}\left(p\right)$ and ${\bar{X}}_{wp}\left(p\right)$ are the local mean intensities over the volume $d^{3}$ of the fixed and warped images, respectively. To achieve a robust evaluation, we analyze patches of varying sizes (ranging from 5 × 5 × 5 to 11 × 11 × 11 voxels) around each voxel. This multi-scale approach enables us to capture both fine details and broader structural features, thereby enhancing the accuracy of the registration. The regularization loss is designed to promote smoothness in the deformation field. We express the regularization loss as:

(4) $$L_{smooth}=\sum_{p\in \Omega }{\left\|\nabla \varphi \left(p\right)\right\|}^{2}$$

Here, ∇φ(p) represents the local gradient of the deformation field, and the loss penalizes significant discontinuities. By minimizing this term, we encourage the deformation to be smooth and continuous, which helps maintain the anatomical integrity of the structures involved. The complete loss function integrates both components as follows:

(5) $$L=-CC\left(X_{fx},X_{wp}\right)+\lambda \sum_{p\in \Omega }{\left\|\nabla \varphi \left(p\right)\right\|}^{2}\;$$

In this formulation, λ serves as a weighting factor that balances the influence of the similarity and regularization losses. By fine-tuning this parameter, we aim to achieve optimal registration and segmentation accuracy while ensuring that the transformations applied are smooth and mostly invertible. Importantly, both terms of the loss functions are computed on the final outputs of the networks, ${X_{wp}}_{n}$ and φ respectively, ensuring a synergy between the cascades

#### 3.1.1 Contracted architecture

One of the main challenges of using cascaded networks is that they can become resource-intensive as the number of cascades increases. To address this issue, we adopted an alternative designed to reduce memory consumption (Fig. 1b and c). This alternative architecture presents higher number of feature maps for the hidden layers, enabling to encode more information for a marginal increase in resources, and it eliminates the final convolution layer on the full-sized image, leading to a substantial decrease in memory consumption. Those modifications enable to achieve equivalent performance while consuming fewer memory resources, as later discussed in Section 3.1.

### 3.2 Multi-atlas segmentation

**Fig. 3:**
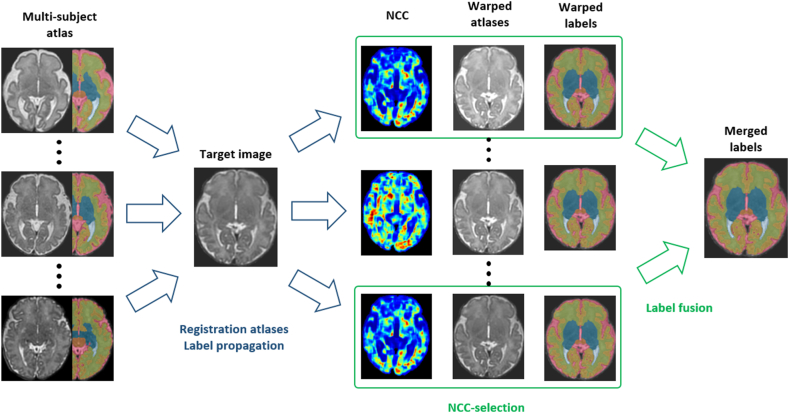
Overview of the MAS method. Registration: all atlases are registered with the target image, with their labels propagated accordingly. Atlas selection: the best-aligned atlases are chosen based on their normalized cross-correlation (NCC with the target image. Label fusion: the selected propagated labels are comined together using local weighted voting (LWV) to generate the final labels.

MAS can be divided into four main steps: image registration, atlas selection, label propagation and label fusion. During the image registration step, each atlas is aligned with the target image using a registration algorithm. This step is crucial because it allows accurate propagation of the atlases labels onto the target image. The atlas selection step involves choosing which atlases to use based on factors such as the similarity between the atlases and the target image before or after registration, and the relevance of the atlases to the specific task at hand. By combining multiple atlases and carefully selecting the most suitable ones, it is possible to achieve improved segmentation accuracy and efficiency. During the label propagation step, the labels from the selected atlases are transferred onto the target image using the DFs obtained from the image registration step. Finally, in the label fusion step, the propagated labels are combined to create the final segmentation of the target image. Based on our cascaded registration model, we propose a simple MAS pipeline for fetal brain MRI segmentation. This approach involves using a subset of annotated images, which are used as atlases and registered to the target image. This multi-subject atlas is composed of 20 subjects between 28 and 44 gestational weeks, that have been selected within the cohort to maximize variability in terms of age, brain development and shape [28]. The best aligned atlases are then selected based on their image similarity with the target image, ensuring that the most suitable atlas images are used to construct the final segmentation. Finally, we propagate the labels of the selected atlases and we combine them using a local weighted voting (LWV) strategy. The overview of the proposed MAS method is shown on Fig. 3.

#### 3.2.1 Atlas selection

The main motivations for selecting specific atlases for use in MAS are, on one hand, to use the more suitable atlases for the task at hand, discarding the ones that overly differ in termsof appearance or morphology, and that ultimately may lower the accuracy of the segmentation, and on the other hand to reduce the computational time. Different similarity measures can be used for atlas selection, such as NCC, Mean Square Error (MSE), and Structural Similarity Index Measure (SSIM). We evaluated those similarity measures and chose the most suitable one for the atlas selection in Section 3.3.

#### 3.2.2 Label fusion

Label fusion is the last critical phase of MAS; it consists of combining the propagated labels from the atlases in order to generate a refined segmentation of the target image. The most straightforward method is Majority Voting [32]. This strategy consists of selecting, for each voxel, the label that is most commonly assigned among all the propagated labels. While this approach is simple and easy to implement, it does not take into account the local similarity between the transformed atlases and the target image, possibly resulting in a less accurate segmentation. In this work, we adopt a technique of label fusion based on LWV [33], which assigns more weight to labels that correspond to areas of high local similarity between the transformed atlases and the target image. By taking local similarity into account, the resulting segmentation is more accurate and better capture the complex structure and variability within the target image. LWV assigns a weight w to each voxel i of the propagated label k as follows:

(6) $$\omega_{k,i}={\left|m\left(i\in \Omega \right)\right|}^{g}$$

where ω is a region of size $d^{3}$ around the voxel i, m is the average local similarity metrics in the region ω, and g is the gain factor, which can take different values depending on thesimilarity metrics used.

### 3.3 Evaluation

Let $Y_{mv}$ and $Y_{fx}$ be the ground-truth labels of the moving and fixed images, respectively. The propagated labels of the moving images are formed by applying a DF φ to the labelsof the moving image $Y_{mv}$:

(7) $$Y_{wp}=Y_{mv}\circ \varphi \approx Y_{fx}$$

We evaluate the performance of our registration model and MAS pipeline using the Dice score [34], and the Hausdorff Distance. The Dice score between the labels Yfxand Ywp is given by:

(8) $$DSC=2\cdot \frac{\left|Y_{fx}\cap Y_{wp}\right|}{\left|Y_{fx}\right|+|Y_{wp}|}$$

The one-sided Hausdorff Distance (HD) from Yfx to Ywp is defined as:

(9) $$hd\left(Y_{fx},Y_{wp}\right)=\underset{y_{1}\in Y_{fx}}{\max\;}\min_{y_{2}\in Y_{wp}}{\left\|y_{1}-y_{2}\right\|}^{2}$$

and from Ywp to Yfx:

(10) $$hd\left(Y_{wp},Y_{fx}\right)=\underset{y_{2}\in wp}{\max\;}\min_{y_{1}\in Y_{fx}}{\left\|y_{1}-y_{2}\right\|}^{2}$$

The bidirectional HD is then:c

(11) $$HD\left(Y_{fx},Y_{wp}\right)=\max\left(hd\left(Y_{fx},Y_{wp}\right),hd\left(Y_{wp},Y_{fx}\right)\right)$$

We opted to evaluate our model using the 95th percentile of the HD, denoted HD95, because it provides a more robust measure of label alignment, by discarding the worst-case errors. All our results are given with the associated standard error, given by:

(12) $${\bar{\sigma }}_{x}=\frac{\sigma }{\sqrt{n}}$$

Where n is the size of the sample, and σ its standard deviation.

### 3.4 Experimental settings

The model was developped using Python 3.8, Pytorch 1.11, and a GPU Nvidia Titan Xp with 12 GB of memory. The baseline architecture of the cascaded networks is a contracted version of Balakrishnan et al. [12], designed to limit memory usage (see Section 2.1). The networks were trained for $EP_{\max}=500$ epochs with 100 iterations per epoch using the Adam optimizer, an exponentially decaying learning rate $lr_{EP}={3\cdot 10}^{-4}\cdot e^{3EP/EP_{\max}}$ and a batch size of 2. Our experiments were conducted using two fetal brain MRI datasets:

• The IMPACT dataset [28,35]: composed of 170 fetal brain MRI scans between 32 and 39 gestational weeks. The single-shot fast spin-echo T2-weighted were performed in Hospital San Joan de Deu (3T Philips Ingenia MR scanner), and Hospital Clinic (3T Siemens Magneton Vida MR scanner). The low resolution stacks were reconstructed using the Ebner et al. [6] super-resolution reconstruction (SRR) pipeline. All fetuses included in this study did not have any major malformation. The dataset is split into 140-10-20 for training, validation and test sets, respectively.
• The FeTa dataset [36]: publicly available dataset composed of 160 T2-weighted fetal brain MRI scans between 20 and 35 gestational weeks, including neurotypical and pathological subjects. The scans were collected from four institutions: University Children's Hospital Zurich (1.5T and 3T GE scanners), General Hospital Vienna/Medical University of Vienna (1.5T and 3T Philips scanners), Lausanne University Hospital (1.5T Siemens scanner), and University of California San Francisco (3T GE scanner). High-resolution 3D volumes were reconstructed using four different super-resolution methods ([6,7]; Dong et al., 2016; [37]). The dataset is split into 130-10-20 for training, validation and test sets, respectively.

The seven anatomical labels are included for the validation and test sets (although they are not required for the validation set, as the weights of the model are saved solely based onthe image similarity loss): cerebro-spinal fluid (CSF), cortical grey matter (CGM), white matter (WM), ventricles (VTC), cerebellum (CRB), thalamus (THA), and brain stem (BS). As a preprocessing step, the images were cropped to remove the non-brain regions, resized to 128 × 128 × 128 voxels, and normalized between 0 and 1.

## 4 Results

### 4.1 Cascaded registration

In this section, we share the outcomes of our experiments concerning the development and optimization of our cascaded registration model. We start by addressing the regularizationof the DF, which plays a crucial role in minimizing voxel folding by promoting a smooth and reversible transformation. Following this, we present the results linked to the contractedarchitecture that serves as a baseline network for the cascades. We showcase how this architecture optimizes memory utilization, enabling the stacking of additional cascades and resulting in overall performance improvements. Subsequently, we introduce the multi-resolution similarity loss and demonstrate its beneficial impact on the registration process. Lastly, we present the results obtained from various iterations of our model, compared with three state-of-the-art DL registration methods [12,14,16].

#### 4.1.1 Quality of the transformation

Ideally, the transformation that aligns the moving image with the fixed image should be smooth and invertible. Non-invertible transformations often contain multiple regions offolding, which can be quantified by the percentage of negative determinant of the transformation's Jacobian. One way to reduce the amount of folding is to use diffeomorphic registration, although this method is generally associated with lower registration accuracy. A key factor in minimizing folding is the regularization parameter λ in Equation (6), which helps to ensure smooth deformation by minimizing the average local gradient. By selecting the value of λ, it is possible to balance the need for smooth deformations with the accuracy ofthe registration. Table 2 shows the percentage of negative Jacobian determinant of the transformation for different values of λ, as well as the average Dice score and HD95 obtained. Based on these results, we adopted a regularization parameter of λ = 1, which provides the best evaluation metrics and significantly reduces the percentage of negative Jacobian values. Fig. 5 illustrates the effect of the regularization parameter λ on the smoothness of the DF. The figure includes a warped image with a grid overlay, a map of the determinant of the Jacobian, and a zoomed-in view of two folding regions for different values of the regularization parameter. It visually demonstrates that higher values of λ lead to smoother DFs and fewer regions of voxel folding (i.e., regions with negative Jacobian determinant).

**Table 2:** Average Dice scores and percentage of negative Jacobian determinant for different regularization parameters.

λ	%|Jϕ|<0	Dice	HD95
10–4	0.458 ± 0.167	0.849 ± 0.021	1.87 ± 0.12
10–1	0.178 ± 0.091	0.859 ± 0.020	1.81 ± 0.11
1	0.062 ± 0.021	0.866 ± 0.020	1.70 ± 0.08
2	0.012 ± 0.003	0.52 ± 0.026	1.75 ± 0.08

#### 4.1.2 Contracted architecture and number of cascaded networks

**Fig. 4:**
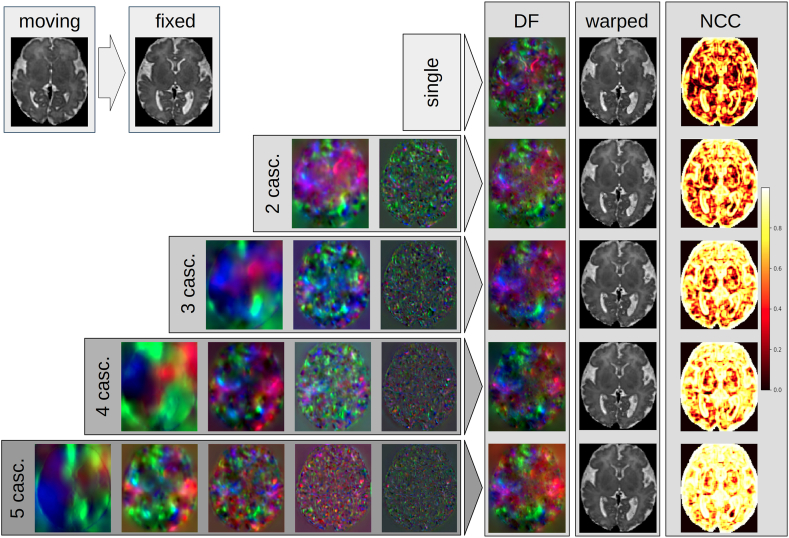
Example of the registration of a pair of images with different numbers of cascades. The multi-resolution DFs are summed together to form the final DF and warp the moving image. The maps of the local NCC between the fixed and warped images demonstrate the better alignment with the increasing number of cascades.

As explained in Section 2.1, we developed a modified version of the VoxelMorph [12] architecture in order to reduce the memory cost and enable the use of more cascades. This modified architecture is referred to as the contracted architecture. Table 3 presents the average Dice scores and HD95 obtained with both the original Voxelmorph architecture and the contracted architecture for different numbers of cascades, as well as the GPU usage during inference. The results presented in the table demonstrate that, while the contracted architecture yields slightly lower scores for a given number of cascades in comparison to the original architecture, it enables the utilization of more cascades due to the reduced memory cost, resulting in enhanced overall performance. We also observe that the percentage of negative Jacobian values decreases with the increasing number of cascades for both architectures. This suggests that the cascades are able to decompose the transformation field into smaller and simpler transformations, which are more likely to be invertible. To visually illustrate the effect of varying number of cascades, we provide an example in Fig. 4, which showcases the registration of an image pair using different number of cascaded networks. The multi-resolution DFs are aggregated to construct the finalDF, which is then applied to warp the moving image. The accompanying maps plot the local NCC between the fixed and warped images, visibly highlighting improved alignment asthe number of cascades increases.

**Table 3:** Average Dice scores, HD95, percentage of negative Jacobian determinant, and memory usage for the original VoxelMorph architecture and the contracted architecture, for different number of cascades. The last two rows for the original architecture are empty because GPU's memory was maxed out.

	Original architecture				Contracted architecture			
N cascades	Dice	HD95	%|Jϕ|<0	GPU(GB)	Dice	HD95	%|Jϕ|<0	GPU(GB)
1	0.818 ± 0.028	2.11 ± 0.14	0.223 ± 0.067	4.6	0.805 ± 0.029	2.15 ± 0.17	0.243 ± 0.096	3.3
2	0.844 ± 0.023	1.98 ± 0.13	0.156 ± 0.045	7.9	0.836 ± 0.023	2.05 ± 0.15	0.168 ± 0.057	4.5
3	0.856 ± 0.021	1.89 ± 0.13	0.100 ± 0.036	11.1	0.854 ± 0.021	1.90 ± 0.12	0.110 ± 0.043	5.6
4	–	–	–	>12	0.859 ± 0.020	1.76 ± 0.09	0.072 ± 0.032	6.7
5	–	–	–	>12	0.866 ± 0.020	1.70 ± 0.08	0.062 ± 0.021	8.1

**Fig. 5:**
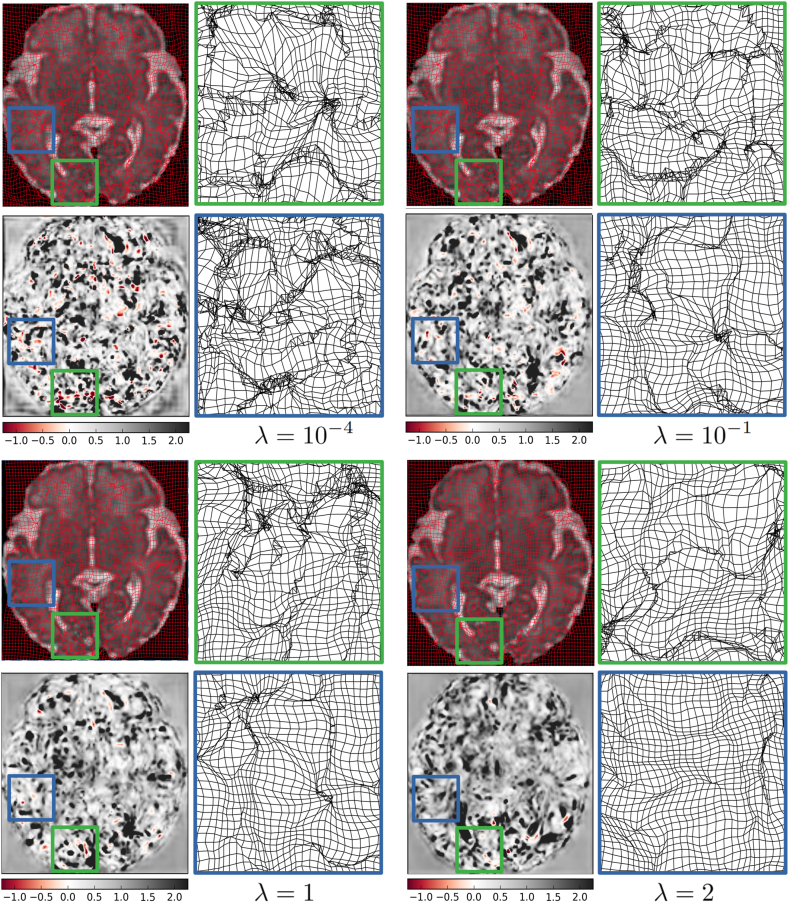
Example of a warped image with different regularization parameter λ, with the deformation grid superimposed, the corresponding Jacobian determinant map and a zoom of the deformation grid on two regions.

### 4.2 Multi-resolution loss

**Fig. 6:**
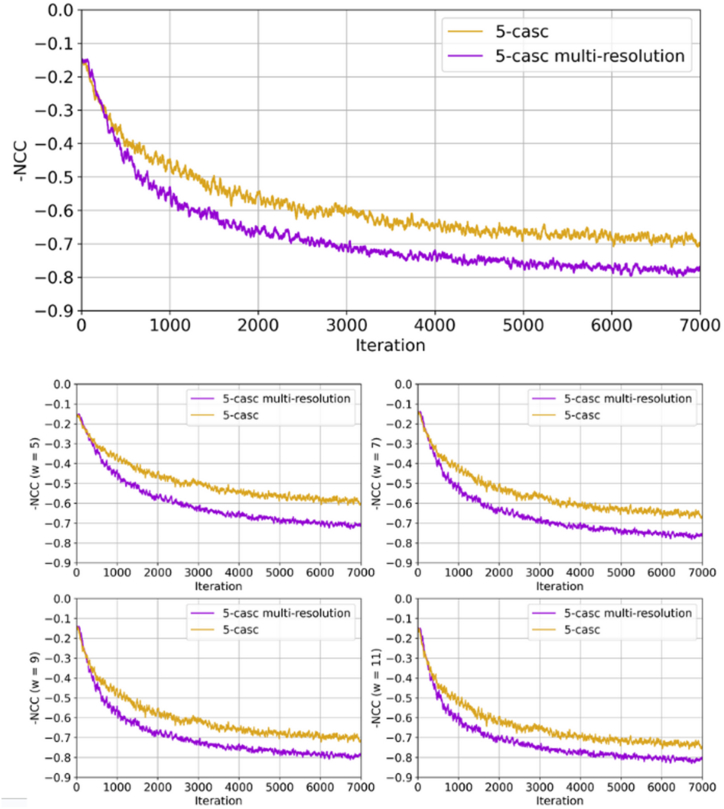
Evolution of the negative NCC during training for a standard 5-cascade model (gold) and a 5-cascade model with multi-resolution image similarity loss (purple). The subplots show the evolution of the negative NCC when computed over different patch sizes (d = 5, 7, 9, 11). On the top, the value on the negative NCC is computed by averaging the multi-scale values.

As elaborated in Section 2.1, the loss function is primarily driven by the NCC (Eq. (4)), which promotes similarity between the warped image generated by the model and the fixedimage. To further encourage the cascaded networks to engage in multi-resolution image analysis, we use an average similarity measure (NCC) over patches of different sizes, with the objective of minimizing image dissimilarities across a spectrum of scales. Fig. 6 illustrates the progression of the image similarity loss, during the training of two 5-cascade networks. In the first network, training relies on an NCC loss with a single patch size of d = 9, while the second network incorporates multiple patch sizes, specifically d = 5, 7, 9, 11. The bottom part of the figure showcases evolution of average local negative NCC for each patch size, highlighting a significant gap between the two methods, with the difference becoming more pronounced for smaller window sizes. On the top plot, the overall NCC, computed by averaging the local negative NCC values across all patch sizes, reveals an improvement of approximately 10 % in the multi-resolution version.

#### 4.2.1 Registration accuracy

**Fig. 7:**
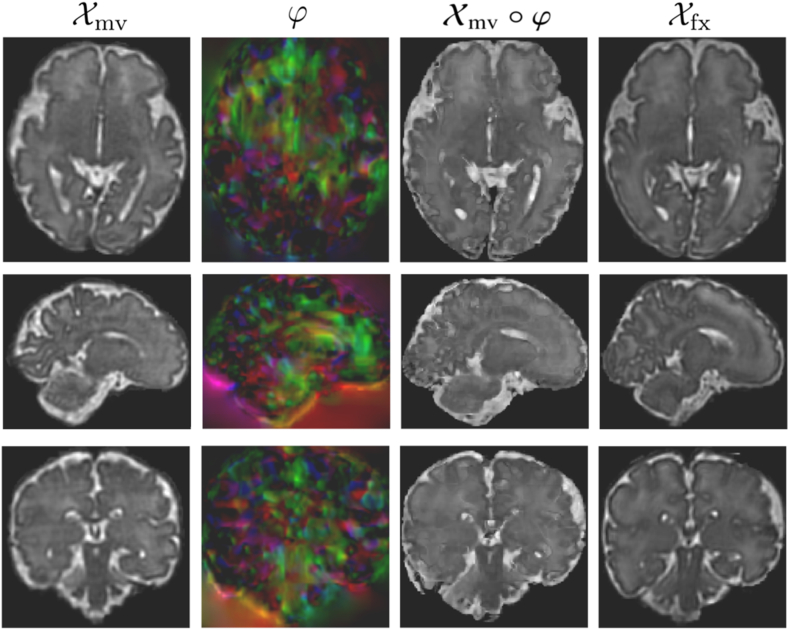

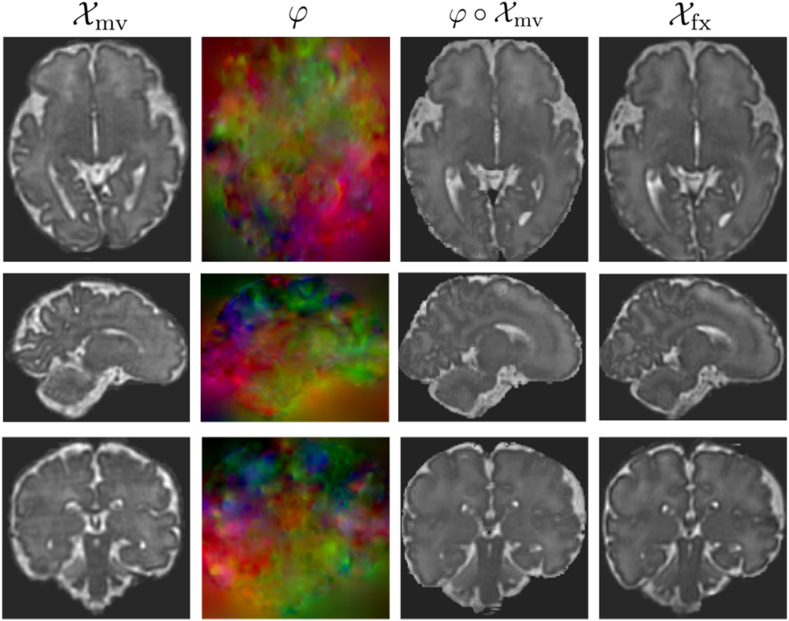
Comparison of the qualitative results of VoxelMorph (top) and our proposed method (bottom). The axial, sagittal and coronal views of the moving, warped and fixed images are shown, as well as the corresponding DF.

Following the cascaded registration method presented in Section 2.1, we evaluate three versions of our registration model on the IMPACT dataset, and compare the results with three state-of-the-art DL registration methods. Specifically, ”CasReg” represents a 5-cascade network incorporating the accumulation of DFs through the cascades. Conversely, ”CasReg-NA” (No Accumulation) warps the moving image successively, with regularization computed on the composition of successive DFs. ”CasReg-MR” (Multi-Resolution) combines the accumulation of the DF with a multi-resolution image similarity loss. Quantitative assessments after registering a consistent set of 50 distinct image pairs are detailed in Table 4, which includes the average Dice scores and H95 of the propagated labels with the ground-truth labels of the fixed images for all seven anatomical structures. It is worth noting that our network was trained to maximize image similarity between the fixed and warped images using trilinear interpolation, while propagated labels were generated by warping the moving image's labels with φ using nearest neighbour interpolation. The evaluation metrics are presented alongside results obtained using VoxelMorph [12], TransMorph [14], and DLIR [16]. Additionally, the Dice score and HD95 after rigid registration are provided for reference. Notably, ”Cas-Reg” outperforms the other methods significantly, achieving an average Dice score of 0.837 ± 0.021 and an average HD95 of 1.81 ± 0.08. The accumulated strategy surpasses these results by a substantial margin, obtaining an average Dice score of 0.866 ± 0.020 and an average HD95 of 1.70 ± 0.08. Furthermore, the multi-resolution version ”CasReg-MR” exhibits additional improvement, with an average Dice score of 0.878 ± 0.021 and an average HD95 of 1.61 ± 0.08. Although the error ranges for the different variants of our method overlap in terms of Dice score and HD95, they do not intersect with the results of the other registration methods tested. This observation underscores the superior registration performance of our approach. Furthermore, paired t-tests were conducted among all methods to establish statistical significance, revealing that our method demonstrates statistically significant differences compared to each of the other methods. Additionally, all three variants of our method exhibit statistically significant differences between themselves. A comparison of the qualitative results of our method with VoxelMorph [12] is shown in Fig. 7. It clearly establishes that the cascaded registration produces better and more realistic results. The appearance of the DF is also substantially different for both methods. This is a consequence of the cascaded approach, which computes recursive DFs at different scales, rather than a single, global DF. Hence, the final transformation is able to capture more accurately the local variations in the shape and structure of the fixed image.

**Table 4:** Average Dice scores and HD95 obtained using ANTs rigid registration [13], VoxelMorph [12], TransMorph [14], DLIR [16], 2 × 10 VTN [17] and the proposed method, with and without DF accumulation.

	Rigid		VoxelMorph		TransMorph		2 × 10 VTN		DLIR		CasReg-NA		Casreg		CasReg-MR	
Labels	Dice	HD95 (mm)	Dice	HD95 (mm)	Dice	HD95 (mm)	Dice	HD95 (mm)	Dice	HD95 (mm)	Dice	HD95 (mm)	Dice	HD95 (mm)	Dice	HD95
CSF	0.516 ± 0.008	4.06 ± 0.16	0.802 ± 0.007	2.35 ± 0.08	0.796 ± 0.009	2.46 ± 0.07	0.801 ± 0.07	1.67 ± 0.07	0.777 ± 0.012	1.99 ± 0.19	0.823 ± 0.007	1.76 ± 0.07	0.855 ± 0.008	1.87 ± 0.06	0.863 ± 0.005	1.84 ± 0.07
CGM	0.501 ± 0.003	3.63 ± 0.17	0.724 ± 0.009	1.69 ± 0.04	0.725 ± 0.011	1.78 ± 0.06	0.646 ± 0.05	1.32 ± 0.05	0.591 ± 0.025	2.58 ± 0.30	0.754 ± 0.009	1.35 ± 0.04	0.781 ± 0.006	1.28 ± 0.04	0.802 ± 0.006	1.26 ± 0.04
WM	0.663 ± 0.003	3.98 ± 0.17	0.782 ± 0.005	2.44 ± 0.07	0.780 ± 0.007	2.57 ± 0.08	0.779 ± 0.02	1.95 ± 0.05	0.638 ± 0.026	3.14 ± 0.25	0.823 ± 0.007	2.00 ± 0.05	0.850 ± 0.004	1.94 ± 0.04	0.853 ± 0.003	1.97 ± 0.05
VT	0.495 ± 0.011	4.37 ± 0.23	0.761 ± 0.017	2.45 ± 0.12	0.751 ± 0.016	2.49 ± 0.15	0.790 ± 0.06	1.81 ± 0.07	0.620 ± 0.023	5.07 ± 1.06	0.812 ± 0.012	1.65 ± 0.09	0.812 ± 0.012	1.63 ± 0.05	0.831 ± 0.008	1.70 ± 0.11
CRB	0.784 ± 0.010	4.67 ± 0.26	0.881 ± 0.009	2.42 ± 0.13	0.873 ± 0.014	2.47 ± 0.10	0.915 ± 0.02	1.62 ± 0.07	0.907 ± 0.012	2.42 ± 0.17	0.899 ± 0.007	1.83 ± 0.07	0.932 ± 0.005	1.85 ± 0.07	0.939 ± 0.003	1.68 ± 0.05
THL	0.812 ± 0.004	3.68 ± 0.18	0.871 ± 0.008	1.82 ± 0.07	0.871 ± 0.007	1.77 ± 0.05	0.895 ± 0.03	1.67 ± 0.06	0.801 ± 0.017	3.80 ± 0.39	0.884 ± 0.007	1.89 ± 0.06	0.915 ± 0.004	1.74 ± 0.04	0.927 ± 0.002	1.61 ± 0.03
BS	0.767 ± 0.005	3.61 ± 0.25	0.856 ± 0.011	1.57 ± 0.10	0.853 ± 0.012	1.56 ± 0.10	0.908 ± 0.03	1.45 ± 0.07	0.868 ± 0.019	1.95 ± 0.16	0.889 ± 0.009	1.78 ± 0.07	0.919 ± 0.004	1.57 ± 0.07	0.928 ± 0.004	1.27 ± 0.04
Average	0.648 ± 0.054	4.00 ± 0.34	0.811 ± 0.023	2.11 ± 0.14	0.807 ± 0.023	2.16 ± 0.15	0.819 ± 0.021	1.64 ± 0.09	0.743 ± 0.018	2.99 ± 0.30	0.837 ± 0.021	1.81 ± 0.08	0.866 ± 0.020	1.70 ± 0.08	0.878 ± 0.021	1.61 ± 0.09

### 4.3 Multi-atlas segmentation

Building on our cascaded registration model, we designed a MAS pipeline and evaluated its performances compared to previous MAS methods for fetal brain MRI segmentation [28,38], and to nnU-Net[30]. Our MAS pipeline relies on the unsupervised training of the cascaded registration model and uses a small subset of 20 annotated images as atlases. This methodology can thus be classified as a weakly-supervised segmentation technique, in contrast to typical DL segmentation methods that draw from extensive annotated datasets. In the following, we first present the atlas selection strategy we opted for, based on the NCC-selection of atlases post-registration. We then discuss the label fusion strategy that we employed to merge to propagated labels into a refined segmentation. Finally, we present our results compared with the aforementioned methods for two fetal brain MRI datasets.

#### 4.3.1 Atlas selection

**Fig. 8:**
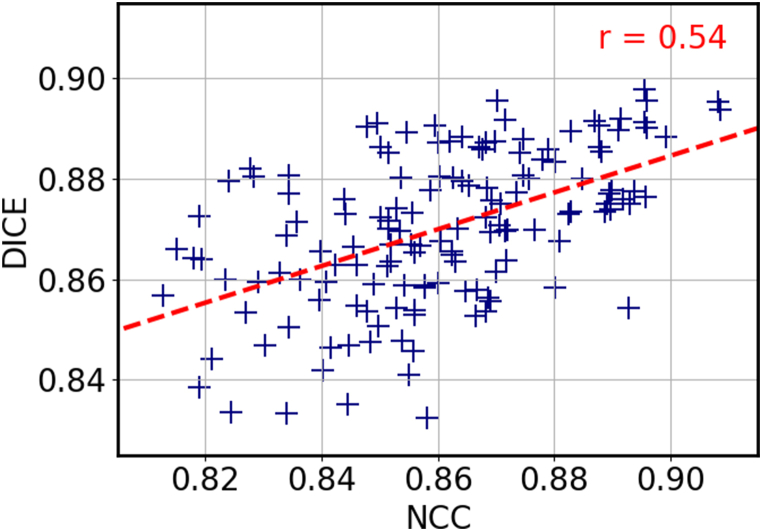

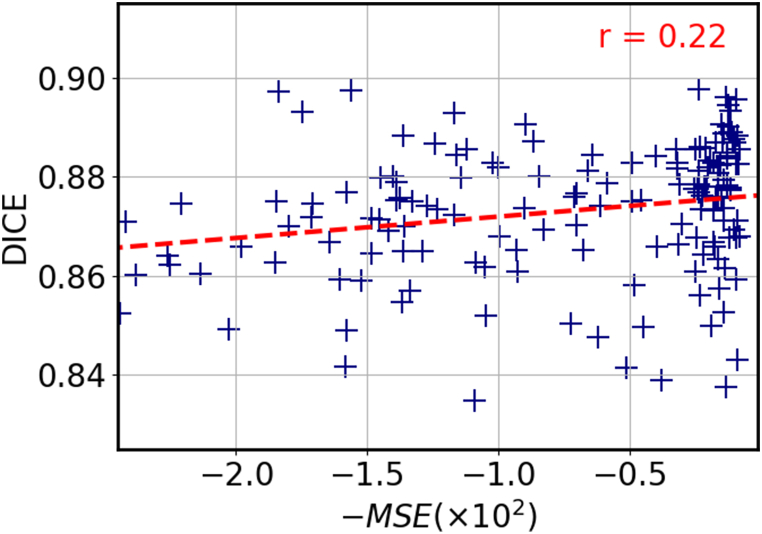

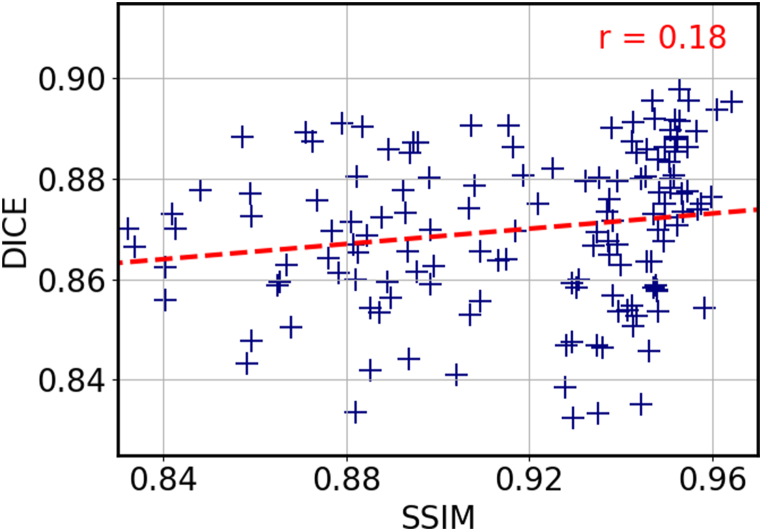

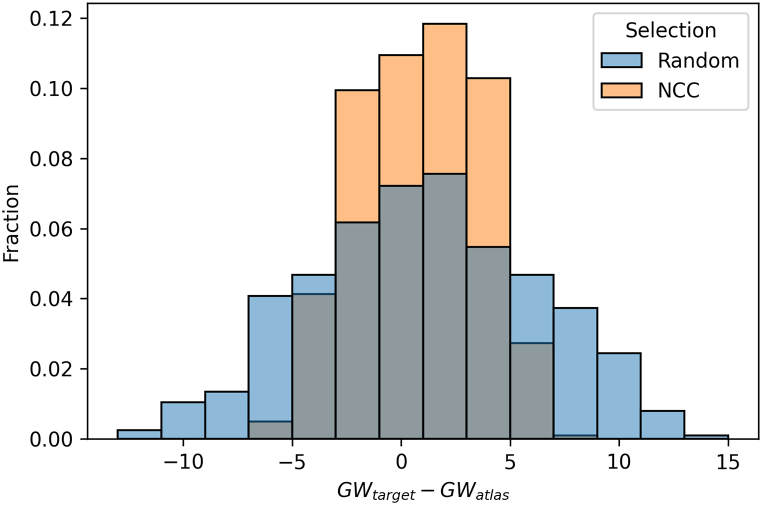
Dice score as a function of the similarity metrics for Normalized Cross Correlation **(a)**, Mean Square Error **(b)**, and Structural Similarity Index Measure **(c). (d)** Histogram of the gestational age difference between the target image and the selected atlases (orange), compared to a random selection (blue).

As mentioned in Section 2.2, the selection of atlases for MAS is an important factor that can affect the accuracy of the segmentation. By carefully choosing atlases that are similar to the target image, it is possible to improve the accuracy of the segmentation by eliminating atlases that are not relevant. With our approach, a pairwise registration is completed in approximately 0.2 s, which allows us to register a large number of atlases with the target image and select the most suitable ones without increasing exceedingly the overall computation time. We examined the effect of atlas selection using three different similarity metrics: NCC, MSE and SSIM. To evaluate the performance of each metric, we calculated the Dice score of the propagated labels and plotted the results in Fig. 8a-c. The figure shows that there is a stronger correlation between higher values of NCC and Dice scores, with a correlation coefficient of r = 0.54, compared with a much lower correlations with r = 0.22 and r = 0.18 for MSE and SSIM, respectively. It is worth noting that these valuesare influenced by the network's training, which was guided by NCC. The outcomes might have varied if an alternative loss function had been employed. Nevertheless, we adoptedNCC as the similarity metric for atlas selection. In addition, we conducted an experiment on the FeTA dataset to further demonstrate the effectiveness of NCC-based atlas selection. The FeTA dataset includes fetuses at various gestational ages, which provides a good opportunity to test the ability of our method to select appropriate atlases, based on the gestational age deviation with the target image. Fig. 8d compares the distribution of gestational age difference between the NNC-selected atlases and the target image to the distribution obtained without selection. Each target image was registered to the other images of the test set, and ten atlases were selected based on NCC in the first case, whereas ten atlases were randomly chosen in the second case. The figure shows that the NCC-selection significantly reduces the age gap between the selected atlases and the target images, compared to the random selection. The selected atlases mostly stand within a range of ±5 gestational week difference from the target image, compared to a much more spread out distribution for the random selection. This result indicates that our atlas selection method not only leads to better alignment of the propagated labels, but also favors atlases that are relatively close in terms of gestational age and morphology.

#### 4.3.2 Label fusion

**Fig. 9:**
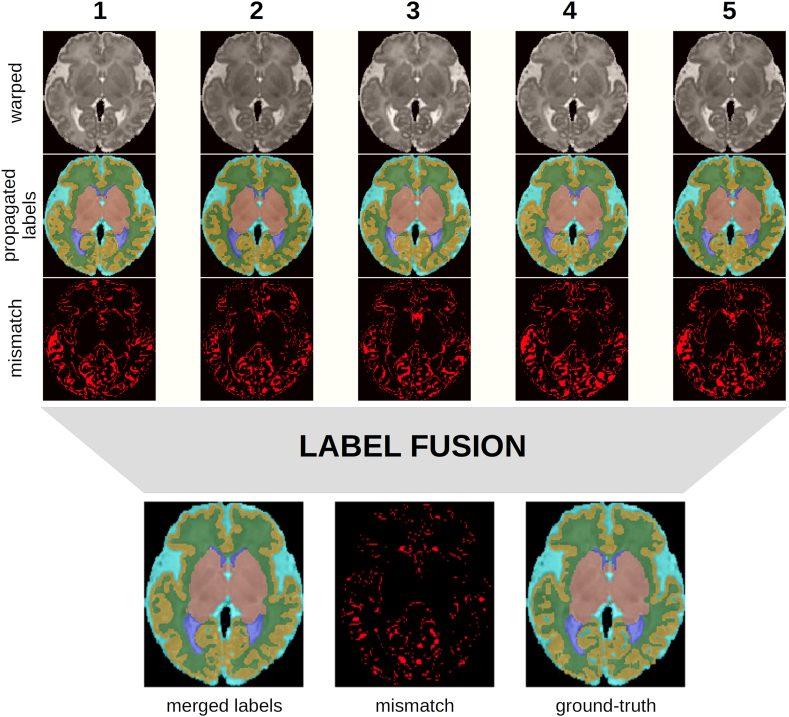
Example of the label selection by LWV. Each column represents one of the NCC-selected atlas, from top to bottom: warped atlas, propagated labels, disagreement between the propagated labels and ground-truth labels. After label fusion, from left to right: final labels, disagreement between the final labels and the ground-truth labels, ground-truth labels.

The label fusion strategy we adopted is based on LWV, as discussed in Section 2.2. LWV, compared to MV, represents a small improvement, with an average Dice score of 0.930 ± 0.012 versus 0.915 ± 0.012, respectively. Fig. 9 illustrates the label propagation and label fusion using LWV. For each propagated labels, a disagreement map with the ground-truthlabels is shown. In this case, the average Dice score over all seven anatomical labels ranges from 0.891 to 0.900 for the propagated labels, and reaches 0.933 after LWV.

#### 4.3.3 Segmentation accuracy

In this section, we present the segmentation results of our MAS method using cascaded registration. We provide a comparison with two classical MAS approaches [28,36] for the IMPACT dataset. Additionally, we compare our segmentation results with nnU-Net [30] for both IMPACT and FeTa datases. For both datasets, a subset of 20 annotated images is used as atlas images, and will be referred as such is the following. The procedure involves first to register each of the atlas images to the target image using the CasReg-MR method detailed in Section 2.1. The ten best aligned atlases are then selected based on the NCC of the warped images with the target image and their corresponding labels are propagated (Section 2.2). Finally, a LWV is applied to combine the propagated labels and form the final segmentation (Section 2.2). Table 5a presents the segmentation results on the 20 fetal brain MRI scans of the IMPACT test set, nnU-Net [30] when trained and tested on the same sets of images, and two classical MAS methods that are the dHCP pipeline for neonatal brain segmentation [38] and the perinatal brain segmentation pipeline [28]. Our method obtains similar results than nnU-Net for all anatomical labels, achieving an average Dice score of 0.930 ± 0.012 and HD95 of 0.87 ± 0.05 versus 0.920 ± 0.014 and 0.95 ± 0.07. The results also demonstrate the superiority of our method compared to classical MAS pipelines for fetal and neonatal brain MRI scans, which obtain an average Dice score significantly lower with 0.87 ± 0.02 for the perinatal brain segmentation pipeline and 0.81 ± 0.03 for the dHCP pipeline for neonatal segmentation. To assess the statistical significance of these results, we conducted paired t-tests between our method and the different comparison methods, considering a p-value of less than 0.05 to indicate statistical significance. The results showed a p-value of 0.102 between our method and nnU-Net, suggesting no significant difference in performance. The comparison with the perinatal pipeline and the dHCP pipeline yielded p-values of 2.17 × 10−2 and 3.99 × 10−2, respectively, indicating a statistically significant difference in favor of our method. The other advantage of our MAS approach when compared to the classical methods is the drastically reduced computational time. For the same single segmentation task, classical approaches require from 30 min up to 1 h, whereas our method segments the image in approximately 60 s, in the same range as nnU-Net. Table 5b showcases the segmentation outcomes on 20 fetal brain MRI scans from the FeTa test set [36], obtained using our MAS method and nnU-Net [30]. In this case, nnU-Net slightly outperforms our approach for 4 out of the 7 anatomical labels, achieving a higher average Dice score of 0.866 ± 0.013 compared to 0.855 ± 0.012 for our method, and HD95 of 3.94 ± 0.36 versus 4.12 ± 0.56, respectively. The comparatively lower performance on the FeTa dataset can be attributed to several factors. Firstly, the FeTa dataset exhibits a greater morphological variability due to the inclusion of fetuses with pathologies that impact the shapes of certain brain regions, such as ventricles in the case of VM. Moreover, the dataset encompasses a broader range of gestational ages, from 20 to 35 weeks, as opposed to the 32 to 37-week range in the IMPACT dataset. Secondly, the FeTa dataset is characterized by a higher degree of heterogeneity as the fetal brain MRI scans were acquired from multiple centers using different scanners and 3D SR techniques, resulting in diverse image appearances within the dataset. This increased variability in morphology and appearance complicates the task of establishing correspondences between image pairs, thereby posing challenges for registration-based segmentation. Although nnU-Net marginally outperformed our method on the FeTa dataset, a notable advantage of our approach is its ability to effectively utilize a limited number of annotated images for MAS, without the need for large amounts of labeled data during network training. This weakly-supervised approach can lead to considerable savings in terms of time and resources, particularly in scenarios where annotated data is scarce.

**Table 5:** (a) Average Dice scores and HD95 obtained using our approach and nnU-Net [30] on the IMPACT dataset. The Dice scores obtained by perinatal brain segmentation pipeline PP [28], and the dHCP pipeline for neonatal segmentation [38], were obtained on the same dataset and are reported from [28]. (b) Average Dice scores and HD95 obtained using our approach, and nnU-Net [30], on the FeTa dataset [36].

	(a)						(b)			
	Ours		nnU-Net		PP	dHCP	Ours		nnU-Net	
Labels	Dice	HD95	Dice	HD95	Dice	Dice	Dice	HD95	Dice	HD95
CSF	0.931 ± 0.006	0.76 ± 0.04	0.897 ± 0.011	1.10 ± 0.20	0.83 ± 0.04	0.79 ± 0.02	0.907 ± 0.008	2.48 ± 0.12	0.878 ± 0.012	3.42 ± 0.18
CGM	0.885 ± 0.006	0.86 ± 0.08	0.871 ± 0.012	1.01 ± 0.09	0.85 ± 0.03	0.76 ± 0.05	0.751 ± 0.009	2.75 ± 0.13	0.762 ± 0.021	2.67 ± 0.12
WM	0.924 ± 0.006	1.00 ± 0.08	0.919 ± 0.006	1.20 ± 0.17	0.90 ± 0.02	0.85 ± 0.02	0.916 ± 0.003	3.32 ± 0.19	0.914 ± 0.006	3.97 ± 0.34
VTC	0.902 ± 0.005	0.83 ± 0.07	0.879 ± 0.011	0.96 ± 0.13	0.73 ± 0.05	0.66 ± 0.07	0.825 ± 0.023	4.76 ± 0.56	0.902 ± 0.014	3.56 ± 0.32
CRB	0.964 ± 0.004	0.99 ± 0.10	0.965 ± 0.004	0.84 ± 0.09	0.93 ± 0.02	0.89 ± 0.03	0.900 ± 0.008	3.47 ± 0.35	0.897 ± 0.008	3.75 ± 0.34
THL	0.952 ± 0.002	0.91 ± 0.06	0.957 ± 0.004	0.85 ± 0.09	0.92 ± 0.02	0.88 ± 0.03	0.859 ± 0.009	6.13 ± 0.83	0.866 ± 0.012	5.67 ± 0.62
BS	0.955 ± 0.003	0.79 ± 0.05	0.953 ± 0.006	0.79 ± 0.05	0.91 ± 0.01	0.90 ± 0.02	0.824 ± 0.006	5.96 ± 0.89	0.841 ± 0.013	4.54 ± 0.45
Average	0.930 ± 0.012	0.87 ± 0.05	0.920 ± 0.014	0.95 ± 0.23	0.87 ± 0.02	0.81 ± 0.03	0.855 ± 0.006	4.12 ± 0.56	0.866 ± 0.013	3.94 ± 0.36

## 5 Discussion

In this work, we introduce a registration model based oncascaded networks and a derived MAS pipeline for fetal brain MRI segmentation. Our registration model decomposes the DF into a series of simpler transformations that operate at different spatial scales, allowing for more efficient and accurate alignment of the atlases with the target image. The key aspects of our approach are on one hand the accumulation of the DFs produced by the cascaded network, which avoids the loss of information that can occur with successive warping and interpolation, and on the other hand the multi-scale image similarity loss that promotes a multi-resolution processing of the images and enable the cascaded networks to capture deformations at different spatial resolutions. Each network focuses on a different level of the registration, from capturing global deformations in the earlier stages to smaller deformations in the later stages. This hierarchical processing helps the networks to capture more complex and non-linear relationships between the images, which are often present in medical image registration problems. To evaluate the performance of our method, we conducted experiments on two fetal brain MRI datasets and compared the results with several state-of-the-art methods [12,14,16]. Our results show that our approach outperforms these methods in both quantitative and qualitative measures. In addition, the derived MAS method achieves performance similar to one of the most robust state-of-the-art segmentation method, nnU-Net [30], despite not requiring any labeled training data. This weakly-supervised segmentation methodology, utilizing a selected subset of annotated data in lieu of traditional atlases, offers a viable alternative for contexts where the amount of annotated data is limited. Another valuable advantage of the MAS approach over DL-based segmentation methods, is its inherent ability to enforce spatial and topological consistency, ensuring smoother transformations and reducing folding or unrealistic distortion in the anatomical structures. This is particularly important in medical image segmentation, where maintaining the anatomical structures and their relationships is crucial for accurate diagnosis and analysis. Furthermore, our MAS method can adapt to different annotation standards and requirements and can be used in multi-center studies, where different centers may have their own specific annotation protocols. It would be therefore possible to incorporate data from multiple centers without the need to standardize the annotation process, potentially saving time and resources. We look forward to exploring further improvements to our framework, particularly through the local regularization of the deformation field by incorporating locally weighted constraints based on biomechanical models. This approach could enhance the accuracy of the spatial transformations, making them more aligned with the underlying anatomical and physiological characteristics of the tissues being analyzed. Future investigations will also focus on analyzing longitudinal data from fetal and neonatal brains. In this context, precise registration is essential for understanding the dynamic changes in brain structures over time and can significantly improve biomechanical models of perinatal brain growth, aiding in early detection of potential developmental issues. Additionally, we will apply our registration framework for the reidentification of nodules in lung CT scans. This application will allow us to track the progression or regression of lung nodules over time, which is critical for effective patient management and treatment planning. We also plan to study cine-cardiac MRI using this registration framework. This will enable us to analyze the dynamics of moving cardiac structures and detect potential pathologies, contributing to a better understanding of cardiac function and disease. Moreover, we aim to adapt and extend our method for various medical imaging applications, particularly in scenarios where obtaining annotated training data is a challenge.

## CRediT authorship contribution statement

**Valentin Comte:** Writing – review & editing, Writing – original draft, Visualization, Validation, Software, Methodology, Investigation, Formal analysis, Data curation, Conceptualization. **Mireia Alenya:** Validation, Resources, Investigation. **Andrea Urru:** Validation, Methodology, Investigation. **Judith Recober:** Resources, Investigation. **Ayako Nakaki:** Investigation, Data curation. **Francesca Crovetto:** Data curation. **Oscar Camara:** Supervision. **Eduard Gratacós:** Data curation. **Elisenda Eixarch:** Data curation. **Fatima Crispi:** Data curation. **Gemma Piella:** Writing – review & editing, Supervision. **Mario Ceresa:** Writing – review & editing, Supervision. **Miguel A. González Ballester:** Writing – review & editing, Supervision.

## Declaration of competing interest

The authors declare that they have no known competing financial interests or personal relationships that could have appeared to influence the work reported in this paper.
